# Racial Differences in Social Capital Volunteer Activities: A Mixed-Methods Longitudinal Study of How Race and Place Influence Opportunities

**DOI:** 10.3390/ijerph23081024

**Published:** 2026-08-05

**Authors:** Ester Villalonga-Olives, Abdolvahab Khademi, Alisha A. Crump, Christopher M. Amissah, Inez Adams, Candace Hall, Yun-Yi Pan, Yusuf Ransome

**Affiliations:** 1 Department of Practice, Sciences, and Health Outcomes Research, School of Pharmacy, University of Maryland, 220 Arch Street, 12th Floor, Baltimore, MD 21201, USA; vahab.khademi@gmail.com (A.K.); aac10277@nyu.edu (A.A.C.); christopher.amissah@morgan.edu (C.M.A.); candace.hall@umaryland.edu (C.H.); yunyipan05@gmail.com (Y.-Y.P.); 2 Graduate Program in Psychometrics, Department of Psychology, Morgan State University, Baltimore, MD 21251, USA; 3 Independent Researcher, Washington, DC 20020, USA; iadamsqualitativeresearch@gmail.com; 4 Department of Social and Behavioral Sciences, Yale School of Public Health, New Haven, CT 06511, USA; yusuf.ransome@yale.edu

**Keywords:** volunteering, volunteer, social capital, social connectedness, measurement invariance, health measurement, longitudinal studies

## Abstract

**Highlights:**

**Public health relevance—How does this work relate to a public health issue?**

- Volunteering, an important dimension of social capital, showed racial and educational differences across three waves of U.S. data.
- These differences matter for public health because volunteering is linked to social connectedness and broader health-related resources.

**Public health significance—Why is this work of significance to public health?**

- The study shows that people do not always define volunteering in the same way, especially when distinguishing formal volunteer roles from informal helping.
- Mixed-methods findings suggest that commonly used survey measures may not fully capture community-based forms of engagement across groups.

**Public health implications—What are the key implications or messages for practitioners, policymakers and/or researchers in public health?**

- Public health research should use more inclusive measures of volunteering that better reflect both formal and informal community engagement.
- Efforts to promote civic engagement should recognize culturally and contextually different ways people contribute to their communities.

**Abstract:**

Volunteering is a key component of social capital associated with improved health outcomes. However, it remains unclear how volunteer patterns differ by race and education and whether standard measures adequately capture racial and contextual differences in how volunteering is understood and reported. This mixed-methods study examined racial and educational differences in volunteer activities over time and evaluated how individuals from different racial backgrounds define, engage in, and value volunteering. This study used a mixed-methods longitudinal design combining secondary analysis of the Midlife in the United States (MIDUS) study (1995–2016; *n* = 5921) with qualitative interviews (*n* = 47) in the United States. The quantitative outcome was the number of self-reported volunteer hours per month across four domains: healthcare, youth-related, political, and other organizations. Linear mixed-effects models were used to examine differences by race, volunteer context, and time, adjusting for age, gender, and educational attainment. Qualitative interviews used MIDUS item probes and open-ended questions to examine participants’ interpretations of volunteer work, unpaid activities, and forms of engagement. Black participants reported significantly higher volunteer hours than White participants (β = 0.97, *p* = 0.007). Volunteering varied by context, with higher engagement in school and youth-related (β = 1.12, *p* < 0.001) and other organizational activities (β = 2.80, *p* < 0.001) and lower engagement in political contexts (β = −0.45, *p* < 0.001), relative to healthcare settings. Volunteering increased modestly over time (Wave 2: β = 0.45, *p* < 0.001; Wave 3: β = 0.39, *p* < 0.001). Interaction analyses indicated that racial differences varied across contexts, with a significantly smaller increase in volunteering within “other” organizations among Black participants relative to White participants (β = −1.29, *p* = 0.006). Qualitative findings indicated that Black participants more often engaged in community-based and informal activities, including work within community organizations, whereas White participants more frequently participated in formal volunteering. Urban residents reported more flexible engagement compared with suburban residents’ structured participation. Volunteering is shaped by structural and contextual factors. Public health research and interventions should adopt more inclusive measures to better capture diverse forms of civic engagement.

## 1. Introduction

### 1.1. Volunteering as a Protective Factor for Health Outcomes

Volunteering has been defined as an expression of individual engagement in activities that are consciously organized and managed [1]. It is unpaid, noncompulsory work done through an organization and for the benefit of people outside the person’s household [2]. Volunteering is an indicator of pro-social behavior, as it is an expression of commitment to a group or collective [3]. Volunteering has been associated with positive health outcomes, especially in older ages [2].

Volunteering is a form of social capital [4,5]. When individuals volunteer, they often engage with others who share similar interests and values, and this can lead to new social connections. Volunteering fosters trust and reciprocity within the community, as people collaborate on shared goals and projects, solidifying bonds within local communities or organizations. Consequently, volunteering enhances social cohesion, nurturing a sense of solidarity and mutual responsibility, while also bridging social divides by uniting individuals from diverse backgrounds. Moreover, volunteering facilitates the exchange of information, resources, and knowledge among participants. Importantly, social capital theorists have distinguished between forms of engagement that generate different types of social capital. In this context, volunteer activities can range from more formal, institution-based engagement to less formal, community-based helping. While formal volunteering can facilitate bridging social capital by connecting individuals across institutional and organizational settings, informal helping behaviors—such as providing unpaid support within one’s community—may more strongly reflect bonding social capital rooted in pre-existing networks of trust and reciprocity.

Volunteering has been advocated by the United Nations, and American and European governments as a way to engage people in their local communities and improve health outcomes [6]. For example, volunteering has acted as a protective factor against risky behaviors such as binge drinking [3]. In a study evaluating whether changes in volunteering are associated with health outcomes, using longitudinal, 4-year data from the Health and Retirement Study, E. Kim et al. showed that participants who volunteered ≥100 h/year (versus 0 h/year) had a reduced risk of mortality and physical functioning limitations, and better psychosocial outcomes [7]. This encompassed higher levels of positive affect, optimism and purpose in life, with lower depressive symptoms, hopelessness, loneliness, and infrequent contact with friends. Similarly, Yeung et al. observed that volunteering had a positive cumulative effect on health outcomes, stating that volunteering should be promoted by public health since it contributes to a healthy lifestyle [8]. Results on volunteering’s positive impact has been further documented at the national level in the 2023 U.S. Surgeon General’s Report, which suggests that volunteering through social programs and within policy frameworks are effective strategies to reduce negative health effects and community isolation [9].

Social capital theory positions civic engagement activities like volunteering as fundamental to building community connections and individual well-being [10]. This theoretical framework conceptualizes volunteering as both an expression and generator of social capital, facilitating the development of trust, reciprocity, and collective efficacy within communities [11]. The networks and relationships formed through volunteer activities create pathways to resources, information, and opportunities that benefit both individuals and their broader communities [12].

### 1.2. Structural Racism and the Impact on Volunteer Activities

Structural racism produces inequalities by directing or constraining what opportunities and resources are available and how they are distributed to people [13,14,15]. This phenomenon is not limited to the actions of individuals; it is deeply ingrained in historical societal, institutional, organizational, and governmental frameworks. This is perpetuated through both formal and informal processes, procedures, and practices that curtail opportunities and resources for specific segments of the population. Structural racism finds support in the prevailing power structures within society and not only shapes community-level access to resources but also directly influences individual behaviors and opportunities, including civic engagement such as volunteering. Disparities in time availability, trust in institutions, exposure to formal volunteer networks, and recognition of informal contributions can all be traced back to structural barriers that disproportionately affect Black individuals [16,17].

The distribution of social capital remains deeply unequal across race and ethnicity. Structural racism theory illuminates how historically entrenched and institutionally maintained systems of oppression have systematically limited marginalized racial groups’ access to crucial social resources. For Black communities in particular, these barriers manifest in restricted access to established social networks, diminished institutional trust stemming from histories of exclusion and discrimination, and fewer pathways to formal civic participation opportunities [18]. These structural inequities shape volunteer experiences in multiple ways. Time and resource constraints disproportionately affect communities facing economic marginalization, limiting their capacity to engage in formal volunteer roles [18]. Additionally, the informal helping behaviors that are prevalent in many Black communities, often go unrecognized as legitimate forms of volunteering by mainstream institutions.

### 1.3. The Need for Qualitative Research to Evaluate Racial Differences in Indicators Assessing Volunteer Activities

In the United States, many indicators to evaluate volunteer activities were developed by White individuals with high socioeconomic status, and some items are tailored to assess situations in predominantly White communities (e.g., volunteering in hospitals). These items may not accurately represent the experiences of Black Americans or those living in predominantly Black neighborhoods. Due to these methodological limitations, there may be questions about the validity of studies comparing levels of volunteer activities and their impact on health outcomes across different racial groups. Consequently, it is essential to evaluate the appropriateness and relevance of the questions used to draw conclusions regarding racial differences in volunteer activities.

Tang et al. observed that Black people are less likely than their White counterparts to volunteer in formal organizations. However, Black people, once volunteering, invest greater time in volunteer activities and benefit more from those activities [19]. J.L. Tavares et al. observed that volunteer activities had a protective effect on hypertension in White people, but not in Black people. They attributed this finding to potential cultural differences related to the meaning of volunteering and contextual differences in volunteering [20]. The discovery by Tavares et al. is pivotal in prompting suspicion regarding cultural differences in volunteer activities that need to be considered when seeking to compare outcomes between Black and White individuals. Our study is based on the premise that cultural differences between Black and White populations exist in volunteer activities. The exploration of these differences is crucial to make recommendations to improve how we measure these differences.

Furthermore, research suggests that when Black individuals are involved in volunteer activities, they are more likely to be involved with at least one community volunteer activity [21,22]. This higher level of engagement could potentially be explained by factors such as religious obligation, strong social networks, and the cultural influence on community involvement within Black communities [23]. The problem is that questions assessing volunteer activities frequently disregard unique experiences of Black people and, instead, operate under the assumption that both Black and White individuals perceive and engage in volunteer activities the same. This oversight fails to recognize the nuanced differences in volunteer experiences between these groups.

### 1.4. Prior Studies That Assess Racial Differences in Social Capital Items

In our previous work, we evaluated the psychometric properties of social capital indicators used in public health research [24]. We found substantial differences across racial groups [24,25]. We suggested that our findings might be influenced by how these items were developed, potentially overlooking the fact that different groups may conceptualize volunteer activities differently. Moreover, certain items may not be equally appropriate for Black and White participants.

To build on these findings, the current study explores whether Black-White differences exist in how individuals report or engage in volunteer activities. We also evaluate the appropriateness of the volunteer-related items used in previous studies, examining their relevance and interpretation across racial groups. Hence, we emphasize the value of qualitative inquiry—particularly through in-depth individual interviews—to gain deeper insights into how volunteering is experienced and understood across groups. This approach is essential to uncover contextual factors that shape volunteer behaviors and to inform the development of more equitable tools for measuring social capital in diverse populations.

### 1.5. The Present Study

In this study, our first aim we will longitudinally examine group differences in volunteering across several settings such as hospitals and political organizations and examine potential Black-White differences in volunteering. We then investigate whether Black-White differences in volunteer activities vary by race (Blacks and Whites) and education. Education is introduced in the analysis because it plays a critical role in shaping opportunities, resources, and social participation, including volunteering. It is also unequally distributed across racial groups due to longstanding structural inequities in access to quality education. Second, examine how individuals from different racial backgrounds (Blacks and Whites) define, engage in, and value volunteering—insights that are critical for developing culturally responsive and equitable social capital measures.

## 2. Methods

### 2.1. Data and Sample

In the present study, we used the questions measuring volunteer activities from the publicly available Midlife in the United States (MIDUS) longitudinal study [26]. MIDUS is an ongoing national longitudinal study led by the University of Wisconsin—Madison and currently funded by the Institute on Aging. The MIDUS study investigates the role of behavioral, psychological, and social factors in age-related variations in the health and well-being of adults aged 25 to 74 in the United States (US). The MIDUS study’s mission is to study health in the US adult population as an integrated biopsychosocial process that unfolds across decades of adult life R [27]. The first wave of the MIDUS study was conducted in 1995/1996 and the sample included 7000 US adults initially aged 25 to 74. In wave II (also called MIDUS 2), conducted between 2004 and 2009, the project was expanded in scope to include study areas such as cognition data and daily experiences and included a new African American sample from Milwaukee, WI. MIDUS 3 was the third wave of the study, which was completed between 2013 and 2016 and included the survey, cognition data and daily experience, among others, and retention-early warning (reinstating participants who dropped out) were added to the project. The MIDUS longitudinal study has demonstrated reasonably high retention, with 77% the overall sample returning for the new round of data collection in wave three, after adjusting for mortality [26]. In the present study, we used data for White and Black participants from waves 1 to 3 of the MIDUS, resulting in a study sample size of 5921 participants.

Table 1 reports the characteristics of our analytic sample. They included 5600 White participants and 321 Black participants. The mean age was 47.3 years (*SD* = 12.9; median = 47; range: 24–93) among White participants and 44.5 years (*SD* = 12.5; median = 44; range: 20–84) among Black participants. Although Black participants were on average slightly younger, the age distributions showed substantial overlap between groups, indicating broadly comparable age composition in the analytic sample. In terms of gender distribution, 47.9% of White participants were male and 52.1% were female, compared to 37.7% male and 62.3% female among Black participants, indicating a higher proportion of females in the Black subsample. Regarding educational attainment, 62.8% of White participants had higher education (college or above) compared to 56.1% of Black participants, while 37.2% of White participants and 43.9% of Black participants were classified as having lower education (high school or less). These distributions suggest modest differences in educational composition across racial groups, with Black participants slightly more represented in the lower education category.

### 2.2. Measures

The questions asking about volunteer activities are part of a subset of the community involvement indicators of the MIDUS main survey. Volunteering among adults is measured by four indicators asking for the number of hours per month the participants volunteered their time in: (1) hospitals or nursing homes; (2) school or other youth related work; (3) political organizations or causes; and (4) other relevant organizations or causes. Responses are measured as the number of hours per month.

### 2.3. Sociodemographic Characteristics

For our study purposes on investigating measurement invariance of volunteer indicators, we selected two sociodemographic characteristics: race (Black and White), and educational attainment (less than high school, GED or HS-Graduate, some college, and college graduate or higher). Educational attainment was used as a proxy for socioeconomic status. In our data analyses, educational levels were dichotomized from the foregoing four groups into low (less than high school and GED or high school graduate) and high level (some college and college graduate and higher). Sociodemographic data were obtained from the first wave of MIDUS data collection.

### 2.4. Statistical Analysis

We examined patterns of volunteer behavior across four contexts: (1) hospitals or nursing homes, (2) schools or youth-related activities, (3) political organizations or causes, and (4) other community settings. Given the longitudinal structure of the MIDUS data, with repeated observations nested within individuals across three waves, linear mixed-effects models (LMMs) were employed as the primary analytic approach. LMMs account for the non-independence of repeated observations by incorporating subject-specific random effects and allow for the inclusion of both time-varying and time-invariant covariates. This approach is more flexible than traditional repeated measures ANOVA, particularly in handling unbalanced data and accommodating participants with incomplete observations across waves.

Monthly volunteer hours served as the dependent variable and were modeled as a function of race (White vs. Black), volunteer context (hospital, school, political, other), and wave (MIDUS 1–3), along with covariates including age, sex, and educational attainment. Education was dichotomized into low (≤high school) and high (college or higher) to facilitate interpretation and address distributional imbalance. Age was included as a continuous covariate given its potential role as a confounder, and sex was modeled as a binary variable (male, female). A race × volunteer context interaction term was included to assess whether racial differences varied across volunteer domains. A random intercept for each participant was specified to account for within-person correlation across waves.

The primary model can be expressed as follows:

Volunteer Hours ~ Race × Volunteer Context + Wave + Age + Sex + Education + (1 | Participant ID)

where Race, Volunteer Context, and Wave were modeled as categorical variables, and Age was included as a continuous covariate.

Model estimates are reported as fixed-effects coefficients (*β*), standard errors (*SE*), and associated *p*-values. To aid interpretation of interaction effects, estimated marginal means (EMMs) were computed for race across volunteer contexts, averaging over other covariates.

Missing data at baseline (Wave 1) were minimal. Overall, approximately 0.4% of data points were missing across all variables. Key demographic variables, including age, sex, and race, had no missing data, while educational attainment had negligible missingness (0.17%). Missingness in the outcome variable (monthly volunteer hours) was slightly higher (4.03%) but remained within acceptable limits. In contrast, missingness in Waves 2 and 3 was primarily attributable to panel attrition (approximately 33%). The use of LMMs allowed for the inclusion of all available observations under these conditions, enabling participants with incomplete data across waves to contribute to model estimation without requiring listwise deletion.

As a sensitivity analysis, we conducted multivariate analysis of variance (MANOVA) to examine group differences in volunteer hours across the four volunteer contexts. While this approach facilitates comparison with traditional methods, it requires complete cases and does not account for within-person variability as flexibly as LMMs. Accordingly, the results from the MANOVA are presented as a sensitivity analysis to assess the robustness of the primary findings.

All LMM analyses were conducted using R statistical software (version 4.5.3), and the sensitivity analyses were conducted in JASP (version 0.19.3.0). Results are presented using a combination of descriptive figures and inferential statistical tables, including mixed-effects model estimates and corresponding estimated marginal means.

## 3. Results

**Figure 1:**
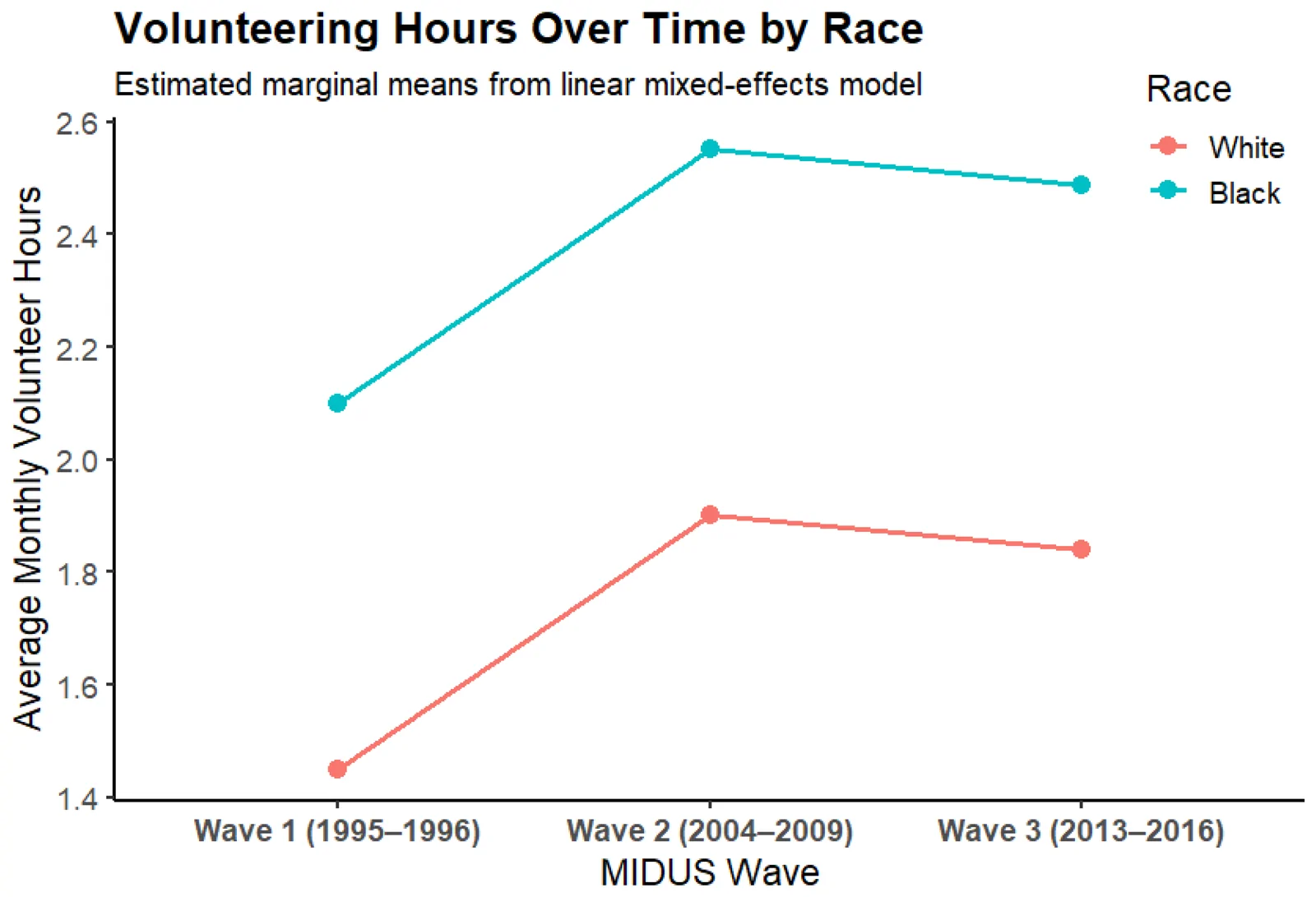
Racial differences in volunteering across three MIDUS waves of White and Black participants.

**Figure 2:**
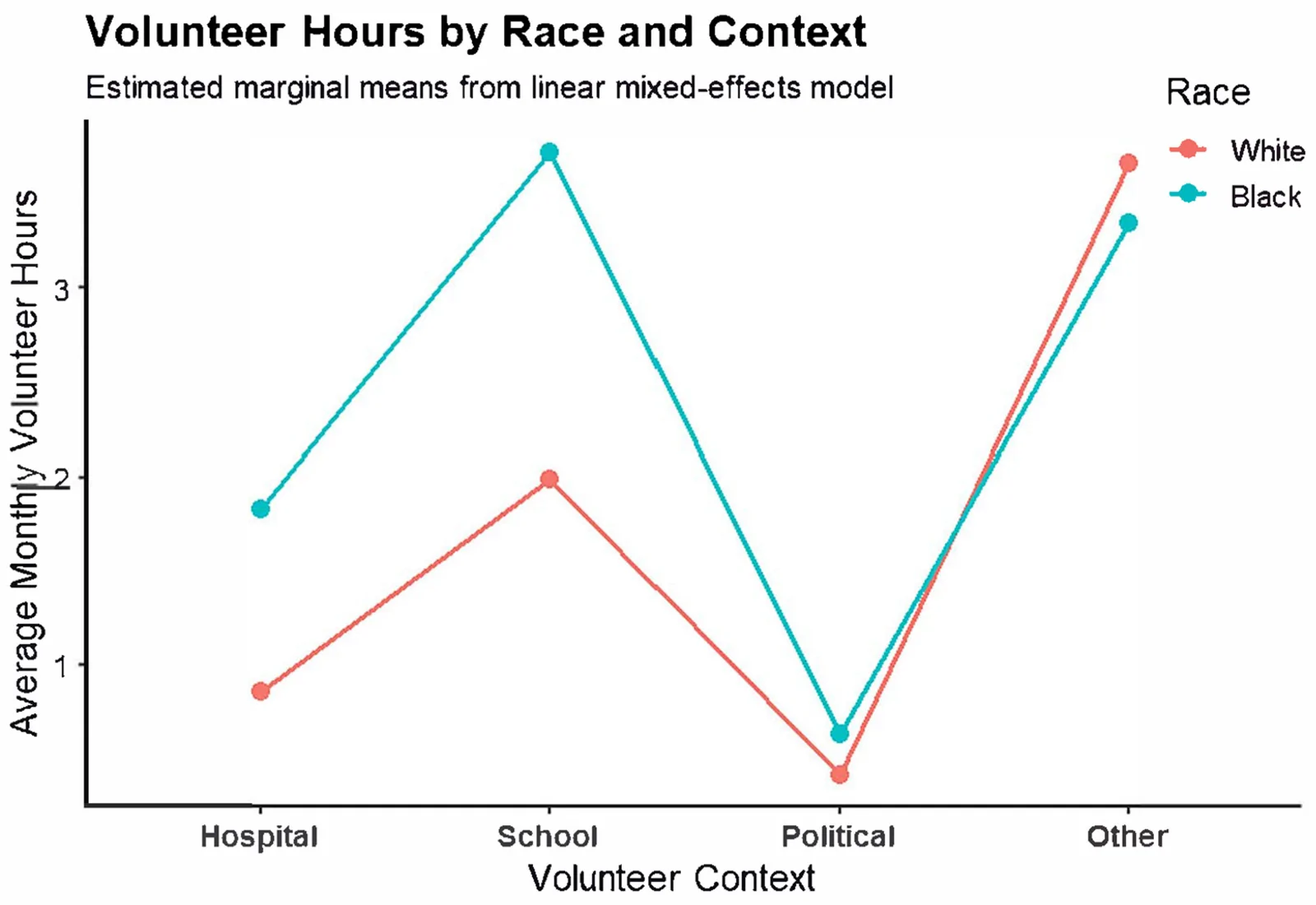
Racial differences in volunteering in hospitals, schools, politics, and other organizational settings averaged across three MIDUS waves of White and Black participants.

**Figure 3:**
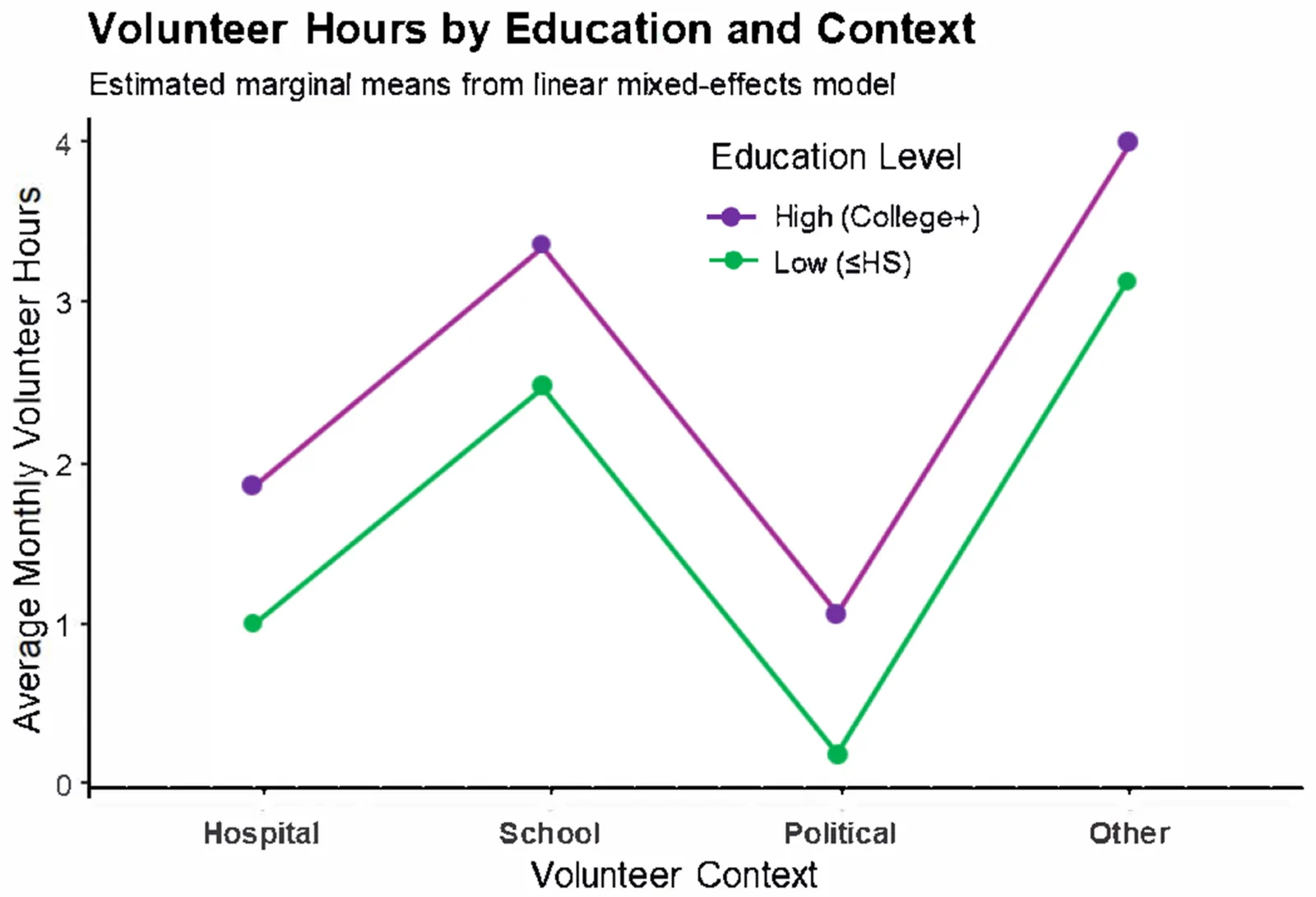
Differences in volunteering by educational level averaged across three MIDUS waves of White and Black participants.

Figure 1, Figure 2 and Figure 3 present descriptive patterns of volunteering across MIDUS waves, volunteer contexts, and educational attainment, based on estimated marginal means from the linear mixed-effects model. As shown in Figure 1, Black participants consistently reported higher volunteer hours than White participants across all three MIDUS waves. Volunteering increased from Wave 1 to Wave 2 for both groups, followed by a slight decline at Wave 3, although levels remained above baseline.

Figure 2 illustrates volunteer patterns across different contexts. Volunteering was highest in “other” organizational settings, followed by school-related activities, hospital settings, and political organizations. The largest racial differences were observed in school and hospital contexts, where Black participants reported higher volunteer hours than White participants. In contrast, racial differences were smaller in political and “other” organizational contexts.

Figure 3 shows differences in volunteering by educational attainment across contexts. Individuals with higher educational attainment consistently reported greater volunteer hours than those with lower educational attainment across all contexts. This pattern was most pronounced in “other” organizational settings and school-related activities, while both groups reported relatively low levels of participation in political activities.

To further evaluate these patterns, results from the linear mixed-effects models are presented in Table 2 and Table 3. As shown in Table 2, Black participants reported significantly higher volunteer hours than White participants (*β* = 0.97, *p* = 0.007). Volunteer varied significantly by context, with higher levels in school-related (*β* = 1.12, *p* < 0.001) and other organizational activities (*β* = 2.80, *p* < 0.001), and lower levels in political contexts (*β* = −0.45, *p* < 0.001), relative to hospital settings. Volunteering also increased modestly over time (Wave 2: *β* = 0.45, *p* < 0.001; Wave 3: *β* = 0.39, *p* < 0.001).

In addition, volunteering was positively associated with age (*β* = 0.017, *p* < 0.001), indicating that older individuals reported slightly higher levels of volunteering than younger individuals. Females also reported higher volunteer hours than males (*β* = 0.29, *p* = 0.002). Lower educational attainment was associated with fewer volunteer hours (*β* = −0.90, *p* < 0.001).

Table 3 further examines the interaction between race and volunteer context. Consistent with the descriptive patterns, racial differences in school (*β* = 0.77, *p* = 0.102) and political (*β* = −0.75, *p* = 0.109) contexts were not statistically significant relative to the hospital reference category. However, the increase in volunteering within “other” organizational settings was significantly smaller among Black participants compared to White participants (*β* = −1.29, *p* = 0.006), indicating that racial differences in volunteering vary across contexts.

Overall, the findings indicate that volunteer behavior differs by race, context, and educational attainment, with persistent racial differences over time and meaningful variation across types of volunteer activities. Importantly, these associations remained robust after adjustment for age, gender, and educational attainment.

### Sensitivity Analysis

To assess the robustness of the primary findings, we conducted supplementary analyses using multivariate analysis of variance (MANOVA) based on raw volunteer hours. The MANOVA results indicated a significant overall effect of race on volunteer behavior (Pillai’s Trace = 0.002, *F*(4, 9704) = 5.49, *p* < 0.001). Follow-up univariate analyses showed that Black participants reported significantly higher volunteer hours than White participants in hospital settings (*F*(1, 9707) = 5.02, *p* = 0.025) and school-related contexts (*F*(1, 9707) = 15.21, *p* < 0.001), while no significant racial differences were observed in political or other organizational settings (*p* > 0.05).

Similarly, educational differences in volunteer were statistically significant (Pillai’s Trace = 0.005, *F*(4, 9704) = 12.63, *p* < 0.001), with individuals with higher educational attainment reporting greater volunteer hours in school-related and other organizational contexts (both *p* < 0.001), but not in hospital or political settings.

Detailed results from these sensitivity analyses are available upon request. Taken together, these findings are consistent with the primary mixed-effects model results, supporting the robustness of observed racial, contextual, and educational differences in volunteer behavior.

To complement these quantitative findings and provide greater insight into the observed patterns, we conducted a qualitative item interpretation substudy. While the MIDUS analyses identify differences in volunteer behavior across race, context, and educational attainment, they do not capture how individuals interpret, experience, or report these activities. The qualitative substudy that starts in the next section was therefore designed to explore how participants understand and describe unpaid and volunteer work, and to provide contextual detail that may help explain the quantitative patterns observed above.

## 4. Item Interpretation Substudy

### 4.1. Study Sample

Participants in this study were recruited via Craigslist.com (accessed on 22 June 2022). Previous studies have reported Craigslist as a viable recruitment method for obtaining participants [28]. Participants were included in the study if they were: (1) age 18 years and above, (2) able to participate in a 1 h video interview, (3) self-identified as Black/African American or White race, (4) had U.S. residency in an urban or suburban location and (5) had a US phone number. Only the first two criteria were included in the Craigslist.com post. To minimize the possibility that individuals who did not meet the desired criteria would enroll in the study, the full list of inclusion criteria was not published on the Craigslist.com recruitment post. The post was published in cities with a significant Black population: Milwaukee (WI), Baltimore (MD), Philadelphia (PA), Atlanta (GA), and San Francisco (CA). However, anyone could view the advertisements and express interest in participating in the study. Therefore, individuals were screened for race and residency status after being contacted by the research team. Individuals with online phone numbers (e.g., Google Voice), were excluded from the study, as an additional effort to ensure that individuals were U.S. residents and humans not bots. The study was approved the University of Maryland Baltimore IRB number HP-00107144.

### 4.2. Data Collection

#### Interview Protocol

The research team created a semi-structured interview guide to identify potential explanations of racial differences observed in the quantitative study and to understand how volunteer activities are experienced and understood when being asked the MIDUS questions. Participants were asked the questions related to volunteer activities that are included in the MIDUS survey: number of hours per month the participants volunteered their time in: (1) hospitals or nursing homes; (2) school or other youth related work; (3) political organizations or causes; (4) other relevant organizations or causes. The intent of asking these questions was to mimic the survey environment by getting participants to provide the responses they would give if they were taking the survey; the intent was not to use the responses to conduct quantitative analyses. Immediately following each survey question, participants were asked open-ended follow-up questions to gather data on their interpretations of the questions and, at times, specific parts of the questions. If participants asked for clarification before providing an answer to the survey question, the moderator redirected them and asked them to respond based on their interpretation. Data were marked as missing if the participants did not provide a response to a given question or if their response fell outside of the defined answer categories. Following the survey questions and follow-up open-ended questions regarding the survey questions, participants were asked how many hours per week they spent doing volunteer work. They were then asked what type of volunteer work they did. Next, they were asked how many hours per week they spent doing things for people outside of their immediate family, for which they were not paid. Follow-up questions were asked to determine if and, when applicable, why they distinguished between “volunteer” activities and “unpaid” activities. Finally, they were shown a list of volunteer activities and asked if they thought there were any common or important activities missing from the list. Interviews were conducted over an online videoconferencing application and lasted between 13 min and 51 min (mean = 29 min and 17 s). Participants received $50 Amazon gift cards for their participation.

### 4.3. Data Analysis

All interviews were transcribed by the research team for analysis. The research team developed a codebook, consisting of broad categories and specific codes within each category [29,30]. Initial codes were informed by the research goals and created prior to transcription analysis. Additional codes were created based on themes that emerged during analysis. The qualitative researcher and research assistant on the team independently coded the first 10 interviews and met to review consensus. The remaining interviews were coded by the qualitative researcher. Data were managed using Atlas.ti (versions 24 and 25) [31].

### 4.4. Qualitative Findings

We present the results of the qualitative analyses in the following order: First, we present the demographic characteristics of the qualitative sample. Subsequently, results are presented from the qualitative interview.

### 4.5. Demographic Characteristics

Table 4 presents the demographic characteristics of the qualitative sample used in the study. A total of 47 individuals completed the qualitative interview. Approximately 39% of participants were 35–44 years old, with over half (55%) identifying as female and White (53%). Most individuals lived in urban areas (66%) and had a total of 1–3 persons living in their household (77%). Household income displayed a wide distribution, with the largest group falling in the $30,000–$39,999 (17%) and less than $30,000 (15%) categories.

### 4.6. Qualitative Interviews

Study participants were asked how many hours per month they spend doing volunteer work and what they understood when asked about it. After asking them to explain their volunteer work activities, they were asked how many hours per month they spend doing activities or errands for others outside of their immediate family, for which they were not paid, to gain more insights about if they thought that volunteer work also involved those type of activities. Black participants reported more hours of unpaid activities beyond their formal volunteer work compared with White participants. The difference between reported volunteer hours and unpaid activity hours was greater among Black participants than among White participants. Black participants were also more likely to report providing professional services without compensation. Specifically, they described using skills and expertise from their professional roles to support individuals and communities. Black and White participants reported engaging in a range of volunteer activities. Common settings included food pantries or soup kitchens, homeless shelters, local schools, and community gardens, with some participants volunteering through places of worship (e.g., churches or synagogues). Participants also noted occasional donations of food and clothing. Although responses did not support clear generalizations about racial differences in the types of volunteer activities, one pattern emerged: Black participants more frequently reported involvement with nonprofit or community-based organizations. In several cases, participants described how their professional roles facilitated additional volunteer engagement or led them to provide assistance to individuals they identified as being in need.

Table 5 presents a selection of quotes resulting from the individual interviews. The majority distinguished between the two and mentioned that unpaid activities often exceeded volunteer hours. The primary reason for the distinction between the two, as explained by the participants, is that they perceived volunteer work to be structured activities managed by organizations or benefiting strangers, while unpaid activities were seen as acts of kindness or necessities for family, friends, or neighbors.

We also observed differences among urban and suburban environments, with participants residing in urban areas more inclined to engage in intermittent volunteer activities, such as distributing clothes or preparing meals for veterans or homeless individuals they personally knew rather than through formal organizations. On the contrary, individuals in suburban areas demonstrated a higher likelihood of reporting regular volunteer work through organized entities. Furthermore, participants from urban neighborhoods often reported engaging in supportive activities for neighbors, such as providing transportation for errands or medical appointments. It is noteworthy that a higher proportion of White participants resided in suburban areas compared to Black participants. Conversely, Black participants were more commonly located in urban areas.

## 5. Discussion

This mixed-methods study found consistent racial and educational differences in volunteering across three MIDUS waves. Quantitatively, Black participants reported higher overall volunteer hours than White participants, with the largest racial gaps in school-based activities. Education was also positively associated with volunteering across most settings. Qualitatively, participants differentiated “volunteer work” (formal, organization-based) from “unpaid activities” (often informal, community-centered), and Black participants more frequently described employment within community organizations and substantial unpaid contributions outside work. These complementary strands suggest both real differences in engagement and potential measurement artefacts when instruments emphasize formal volunteer contexts.

Our study has several strengths. We analyze a large, longitudinal, nationally representative cohort with three waves of harmonized items and triangulate those results with in-depth interviews, offering context for interpreting group differences. We also examine multiple volunteer domains rather than a single composite. Important limitations should be acknowledged. The number of Black respondents is markedly smaller than the number of White respondents, which may reduce the precision and generalizability of racial comparisons. In addition, key characteristics such as size and type of volunteer activities, as well as family or friend influences on participation, were not available in the MIDUS data and therefore could not be included in the analyses. Missing data were handled using complete case analyses, which may bias estimates if data are not missing at random. The qualitative component also has limitations. The interview sample, while diverse, skewed female and urban, and recruitment via online postings may favor individuals with specific experiences or access to resources. Moreover, the interviews did not systematically collect information on barriers to participation or other contextual factors that may influence engagement in volunteer work. Participants who choose to engage in interview-based research may also differ from the broader population, for example by being more comfortable sharing their perspectives or having a greater interest in the topic. While recruitment through an online platform may shape sample characteristics, participants were primarily drawn from urban and suburban areas where digital access is common. Accordingly, findings should be interpreted within the context of the qualitative sample, with emphasis on the range and depth of perspectives rather than generalizability to all populations. Furthermore, because participants were asked to respond to standardized survey questions and then discuss their interpretations, the interview process may have created an artificial research setting that influenced response formulation. Although this approach was intentionally used to explore cognitive processes and reduce retrospective recall bias, participant reactivity and the added cognitive burden of reflecting on one’s reasoning may have affected how some responses were expressed. Finally, our measures capture hours of engagement but do not account for the intensity, social value, or health impact of different forms of informal helping.

In relation to prior literature, our results both align with and extend existing evidence. Earlier studies report that Black adults are less likely to volunteer formally but invest more time when they do and may derive different health returns from formal volunteering than White adults [19,20]. Our findings corroborate higher time investment—especially in school settings—and add qualitative evidence that formal metrics may undercount culturally salient forms of civic contribution (e.g., church-based roles, neighbor support) more prevalent in Black communities [22,32]. The educational gradient we observe is consistent with social capital theory and cross-national work linking education to organizational involvement [33,34]. Together, these patterns indicate that apparent “deficits” in volunteering for some groups may reflect construct under-coverage rather than true lower engagement, particularly where instruments privilege formal organizations.

These findings have several implications for interpretation and practice. First, differences in reported volunteering likely reflect both genuine behavioral patterns and differential item relevance. Instruments that emphasize hospitals, non-profits, and political organizations may insufficiently capture informal, community-embedded helping—work that our interviewees described as regular, relational, and often substantial. For clinicians and health systems, recognizing and partnering with community-defined forms of contribution (e.g., faith-based ministries, mutual aid, school-family networks) could strengthen social prescribing and referral pathways in ways that are culturally responsive. For policymakers and program designers, broadening operational definitions of volunteering, supporting Black-led and community-rooted organizations, and reducing barriers to engagement (e.g., scheduling flexibility, transportation, childcare) may better leverage existing community assets while promoting equity in civic participation and its potential health benefits.

Unanswered questions point to a clear research agenda. Measurement work should prioritize content validity and cultural responsiveness—co-designing items with community partners; distinguishing formal volunteering from unpaid helping while valuing both; and testing longitudinal and cross-group measurement invariance. Future studies should include larger and more diverse samples across racial/ethnic groups, integrate geocoded data to interrogate urban–suburban differences and historical segregation, and examine mechanisms (e.g., institutional trust, work constraints, caregiving load) that shape volunteering trajectories over time [33,34,35,36]. Mixed-methods and quasi-experimental designs, alongside improved metrics, can clarify how different forms of civic contribution relate to health and wellbeing, and which policy levers (education systems, employers, municipalities) most effectively and equitably expand opportunities.

## 6. Conclusions

This study highlights how racial and educational patterns in volunteering are shaped not only by differences in participation but also by how civic engagement is defined and measured. By integrating longitudinal survey data with qualitative accounts, we show that formal volunteer metrics may undercount substantial informal and community-embedded contributions, particularly those described by Black participants. These findings suggest that apparent group differences can reflect construct under-coverage when instruments privilege organization-based activities over unpaid, relational, and community-centered helping. These findings point to the importance of developing more culturally responsive measurement approaches that better capture diverse forms of contribution and more closely align operational definitions of volunteering with lived experience.

## Figures and Tables

**Table 1:** Demographic characteristics of analytic sample of MIDUS Waves 1–3 participants by race, White or Black.

	Analytic Sample (*n* = 5921)	
Variable	White	Black
Race	5600 (94.6%)	321 (5.4%)
Age (years), mean (SD)	47.3 (12.9)	44.5 (12.5)
Sex		
Male	2683 (47.9%)	121 (37.7%)
Female	2917 (52.1%)	200 (62.3%)
Education		
Low (≤High School)	2082 (37.2%)	141 (43.9%)
High (College+)	3508 (62.8%)	180 (56.1%)
Missing	10 (0.2%)	0 (0.0%)

*Note.* Values are number, % except when specified otherwise. Education percentages are calculated among non-missing values; missing education is reported separately.

**Table 2:** Linear mixed effects model results for volunteer hours in analytic sample of White and Black participants from MIDUS Waves 1–3.

Predictor	*B*	*SE*	*p*	Interpretation
Race (Reference: White)				
Black	0.97	0.36	0.007	Black participants reported higher volunteering hours
Volunteer Context (Reference: Hospital)				
School	1.12	0.10	<0.001	Higher volunteering in school-related activities
Political	−0.45	0.10	<0.001	Lower volunteering in political activities
Other	2.80	0.10	<0.001	Highest volunteering in other organizations
Time (Reference: MIDUS Wave 1)				
MIDUS Wave 2	0.45	0.09	<0.001	Slight increase over time
MIDUS Wave 3	0.39	0.11	<0.001	Sustained increase over time
Age	0.017	0.004	<0.001	Older individuals volunteer slightly more
Education (Reference: High education)				
Low education	−0.90	0.09	<0.001	Lower education associated with less volunteering
Gender (Reference: Male)				
Female	0.29	0.09	0.0017	Females volunteered more

**Table 3:** Race × volunteer context interaction and estimated marginal means (hours/month) in analytic sample of White and Black participants from MIDUS Waves 1–3.

Volunteer Context (Reference: Hospital)	Mean Volunteer Hours					
	White	Black	Difference	*B*	*p*	Interpretation
Hospital	0.86	1.83	+0.97	0.97	0.007	Higher among Blacks
School	1.99	3.72	+1.73	0.77	0.102	No statistically significant difference
Political	0.42	0.63	+0.21	−0.75	0.109	No statistically significant difference
Other	3.66	3.34	−0.32	−1.29	0.006	Smaller increase among Blacks

*Note*. Estimated marginal means are derived from the linear mixed-effects model. *β* coefficients for interaction terms represent differences in slopes relative to the reference category (hospital settings). Mean differences are descriptive and do not represent model coefficients.

**Table 4:** Descriptive Characteristics of Qualitative Sample (*n* = 47).

Demographic Characteristics		*N* (%)
Age	18–24 years old	2 (4)
	25–34 years old	8 (17)
	35–44 years old	18 (39)
	45–54 years old	9 (19)
	55–64 years old	6 (13)
	Greater than 65 years old	2 (4)
	Missing	2 (4)
Gender	Female	26 (55)
	Male	18 (39)
	Non-Binary	2 (4)
	Transgender	1 (2)
Race	White, Non-Hispanic	25 (53)
	Black, Non-Hispanic	22 (47)
Population Density	Urban	31 (66)
	Suburban	15 (32)
	Rural	1 (2)
Number of Persons Living in Household	1–3 persons	37 (79)
	4–6 persons	2 (4)
	Missing	8 (17)
Household Income	Less than $30,000	7 (15)
	$30,000 to $39,999	8 (17)
	$40,000 to $49,999	2 (4)
	$50,000 to $59,999	4 (9)
	$60,000 to $69,999	2 (4)
	$70,000 to $79,999	5 (10)
	$80,000 to $89,999	2 (4)
	$90,000 to $99,999	4 (9)
	Greater than $100,000	4 (9)
	Missing	9 (19)

**Table 5:** Results of the individual qualitative interviews (*n* = 47).

Racial differences volunteer work vs. unpaid work	I just felt like that (providing free English lessons) was something that was more on an individual basis and it wouldn’t really enter into volunteer work. It’s more like something that I do out of my heart to help new immigrants to the country. (White female talking about volunteer work) As of right now, I have not done any real volunteer work in a few years… Now, I guess, being an advisor with the department in church, that is not seen as volunteer, but I guess that would be considered because I don’t get paid to do that. I would consider that to be my volunteer, but actually volunteering for something like for Red Cross or something like that, no… I guess, when I think about volunteer work, I think about volunteering for something like the Red Cross or the hospital. I never really thought about work at church as volunteer. I don’t know why and I’ve been doing it for years, but I never thought about it as volunteer work. (Black female talking about doing unpaid activities at the church that she did not consider volunteer work) I’m thinking about my clients. They’ll give their friends my number and be like, “Oh, she can help you with this, this, and that.” I feel bad when they call because I’m like, “You’re not on my caseload. I can’t really service you the way I need to,” but I try to give them the resources I can. I’ll say probably about like 10 h of the month. (Black female talking about unpaid labor she did not consider volunteer work)
Urban–suburban differences	In Las Vegas specifically, we have a lot of homeless individuals and so my husband and I like to spend time every month helping out. Whether it’s with handing out meals or spending time handing out clothes and things like that at one of the shelters, which I actually found out about through my job. We’ll go and spend time there and specifically with some of the adolescents and things like that, so that’s usually what we’ll do every month. (Suburban Black female) We have these Backpack Buddies where we get food and stuff and send them to kids that might not have food or whatever. I volunteered for Relay for Life, that’s a cancer thing. My mom had breast cancer, my dad had pancreatic and prostate cancer, so I’m very involved in cancer things. My daughter, my youngest just had a 5k run about three weeks ago, and we were out there volunteering for her school too, so there’s a lot. (Suburban Black female) I like to volunteer at soup kitchens and food banks. (Suburban Black female) I do clean up around my neighborhood. I just walk around and pick up a lot of litter on my own. (Urban White female) I may take people places who need a ride someplace, take them to a doctor visit. If someone needs to go to a food shop, I’d pick up their food for them. (Urban White female)

## Data Availability

Data is available upon request.
